# Earthquake source parameters in Zagros region (Iran) from the time-evolutive P-wave displacement

**DOI:** 10.1038/s41598-023-45119-x

**Published:** 2023-10-20

**Authors:** Sahar Nazeri, Fatemeh Abdi, Amir Ismail, Habib Rahimi, Aldo Zollo

**Affiliations:** 1 Department of Physics E. Pancini, University of Naples Federico II, Naples, Italy; 2 Institute of Geophysics, University of Tehran, Tehran, Iran; 3 Department of Geology, Faculty of Science, Helwan University, Cairo, Egypt

**Keywords:** Geophysics, Seismology

## Abstract

The rupture process of the recent moderate-to-large earthquakes in the Zagros area along the Iran plateau is investigated by analysing the strong motion data provided by the Iranian Building and Housing Research Centre (BHRC). The selected dataset includes the largest and deadliest 2017 Mw 7.3, Iran-Iraq (Ezgeleh) earthquake. The earthquake source parameters (moment magnitude, rupture duration and length, average slip, and static stress drop) are determined using a time-domain, parametric modelling technique based on the time evolution of the P-wave displacement signals. The earthquake source parameters are calculated from simulated triangular moment-rate functions assuming the circular source models for a constant rupture velocity. The anelastic attenuation effect is modelled through the independent frequency-Q parameter ranging from 50 to 200 and accounted for by a post-processing procedure that retrieves the attenuation-corrected, moment-rate triangular shape. Results show that the average static stress-drop with different ${Q}_{P}$, varies between <Δσ>  = 0.9 (0.7–1.2) MPa and <Δσ>  = 1.6 (1.2–2.0) MPa. Overall, in this research, the rupture radius/length empirically scales with the seismic moment with a self-similar, near-constant stress drop of about 1 MPa. Assuming a circular rupture model for the Ezgeleh earthquake, we estimate a moment magnitude of 6.9, rupture duration of 7 s, source radius of 16 km, average slip of about 2 m and static stress drop of 3.4 MPa.

## Introduction

Iran territory is a part of the Alpide-belt, a very complex tectonic area under the continuous convergence stress acting between the Arabian margin and the Eurasian plate, that is the cause for the shortening and thickening of the continental crust in Iran^1^. A number of studies^2–8^ have classified the Iranian plateau into different seismogenic provinces. Canitez^2^ divided Iran into three main parts as southern, central, and northern based on the fault mechanism characteristics of the occurring earthquakes. According to Nowroozi and Ahmadi^3^, Iran's seismic risk is divided into two main seismic zones (southwestern and northern/eastern Iran) based on the occurrence rate of M 6.0–7.5 earthquakes. Masson et al.^7^ subdivided Iran into two main geological/seismic regions, the southern and northern Iran characterized by aseismic and seismic deformation, based on the observed seismic and geodetic strain rates respectively. Mirzaei et al.^4^ and Tavakoli and Ghafory Ashtiany^6^ proposed to divide Iran into five and twenty seismotectonic zones, respectively. Raeesi et al.^8^ propose the evidence for eleven mature fault segments generating major earthquakes upon the combined analysis of geodetic data and the recent and historical earthquake catalogues.

In spite of the relevant complexity of the seismological research, there is good agreement for one of significant seismogenic areas that lies in the south-west to south region of Iran called the Zagros region. It is a collision zone resulting from a long-standing compressional motion of Eurasia-Arabian plates. The Zagros zone is the most extended seismic sector with the highest seismic activity rate and one of the active fold-and-thrust belts in Iran producing intense and low-magnitude seismicity^1,7^ as well as large and damaging events.

**Figure 1:**
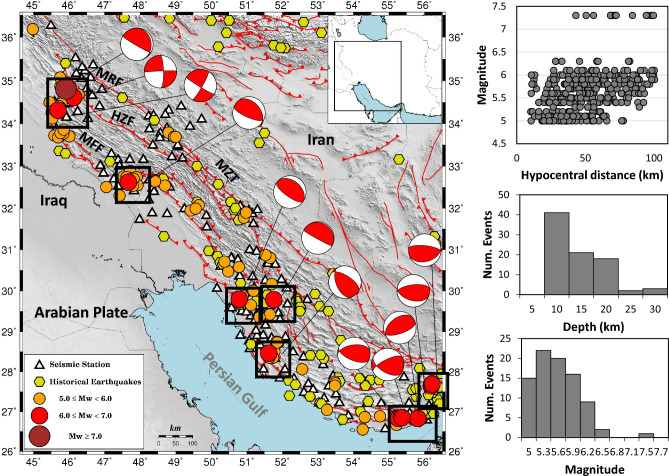
Map shows the locations of the acceleration stations belonging to ISMN (triangles) and epicentres (circles) of the selected earthquakes. The distribution of the focal depth, magnitude, and moment magnitude versus hypocentral distance for the whole database is also shown in this figure. The red lines present the main faults of the region including the Main Zagros Thrust (MZT), Main Recent Fault (MRF), High Zagros Fault (HZF) and Mountain Front Fault (MFF). Segments including large events are shown in rectangles. Available historical events^10^ are shown with yellow hexagons.

The Zagros collision belt built an active seismic zone, i.e., the Zagros region, that extends almost 1500 km from the East Anatolian fault in eastern Turkey to the Makran subduction zone in southern Iran (Fig. 1). This region includes several main faults like the Main Zagros Thrust (MZT), Main Recent Fault (MRF), High Zagros Fault (HZF) and Mountain Front Fault (MFF), where the MZT is a major structural discontinuity that separates the Zagros belt from Central Iran^9^. Historical seismicity catalogue provided by Berberian^10^ in the selected area implies that the region is also known to produce large earthquakes Mw > 5.0 (shown with hexagons in Fig. 1).

In light of the rare occurrence of coseismic ruptures on the surface^11^ and the presence of the Hormuz salt at the base of the folded sedimentary, several previous studies^9,12,13^ concluded that large earthquakes in the Zagros usually occur within basement faults. Interestingly, more recent geodetic studies that used radar interferometry to determine the depth extents of coseismic faults have discovered that moderate earthquakes (Mw, 5 to 6) rupture at depths between 4 and 9 km within the sedimentary cover^14–16^.

The Zagros area is characterized by a low seismic strain rate^8^. The current rate of North–South shortening occurring in Zagros (as recorded by GPS) varies across the range, from a maximum of ~ 9 mm year^−1^ in the SE to ~ 4 mm year^−1^ in the NW^17,18^ which is mainly accommodated by an aseismic deformation ranging from 28 to 39 percent^19^.

In this study, we used the strong motion data provided by the Iranian Building and Housing Research Center (BHRC), belonging to Iran Strong Motion Network (ISMN) to investigate the source characteristics of the recent moderate-to-large earthquakes in the Zagros region. Since 1973, ISMN has been continuously expanded and updated, by deployment of new seismic stations. The network currently consists of 1160 seismic stations mostly equipped with SSA-2 and CMG-5TD 3-component sensors.

The selected dataset includes the records of the 2017 Iran-Iraq earthquake (known as the Ezgeleh earthquake) that occurred in the northwest Zagros fold-thrust belt zone. After the 1909 Mw 7.4, Sialkhor earthquake, the Ezgeleh earthquake is the largest and deadliest earthquake in the Zagros region. It caused 600–700 deaths, 9000 people were injured and thousands left homeless. In addition, the earthquake also triggered a massive landslide to the north of Ezgeleh City^20^. Feng et al.^21^ have proposed a hypocenter at 15 km depth along a basement fault that is causative for the 2017 earthquake in the Zagros region where large earthquakes have not been documented for several centuries. A coseismic source model proposed by Jamalreyhani et al.^22^ suggested an oblique thrust mechanism and a fault plane with a low dip angle in the Precambrian basement. In several studies, two high slip asperities are revealed for this event with a maximum slip of about 5 to 6 m^21,23,24^. Whereas Gombert et al.^25^ using near-source seismological and satellite geodetic data and kinematic slip modelling observed a high impulsive source with a peak slip of 5.5 ± 0.5 m, and southward rupture directivity. In addition, from discrepancy between the coseismic slip direction and the plate convergence, Gombert et al.^25^ concluded existence of the strain partitioning between thrust and unmapped strike-slip faults in this part of the Zagros belt.

Here, the earthquake source parameters i.e., moment magnitude, source duration, source dimension, and average stress drop, are determined applying a parametric modelling technique named the “LPDT-method” (logarithm of the P-wave displacement amplitude vs time) to the near-source strong motion data^26–30^. With the aim of constructing the moment rate function of a given earthquake, this method is based on building and modeling the time-evolution of the P-wave displacement amplitude in a progressively expanded time window, starting from the first P-wave onset using the time step as small as the time-sampling of the data.

This time-domain method has been already validated through the application to the strong motion datasets of the 2016–2017 Central Italy seismic sequence and 2007–2019 Japan earthquakes covering a wide moment magnitude range (Mw 2.5–6.5) and recording distances smaller than 100 km^27,28^. Nazeri and Zollo^29^ developed a MATLAB package named EASOt-AP implementing the LPDT method along with presenting an application to the small magnitude events (2 ≤ Mw ≤ 3) that occurred in Irpinia, southern Italy. Also, the method has been recently applied to the microearthquakes that occurred during fluid extraction/injection around Prati-9 and Prati-29 wells in the Geysers geothermal field in 2007–2014^30^.

Furthermore, in order to overview the regional dependency of the method and measurements, the results of this study are compared with those of Zollo et al.^29^ for Central Italy and Japan. Last but not least, a major objective of this study is to develop empirical scaling laws which can provide valuable insights into fundamental seismic event characteristics, such as rupture dimension, so that seismic hazards can be managed, resilient structures can be built, and early warning systems can be developed effectively as well.

## Data and method

### Data

In this study, we use the acceleration records of about 51 moderate-to-large earthquakes with Mw ≥ 5.0 that occurred in Zagros in the period 2006–2022 for which corresponding records pass the data quality checks.

**Figure 2:**
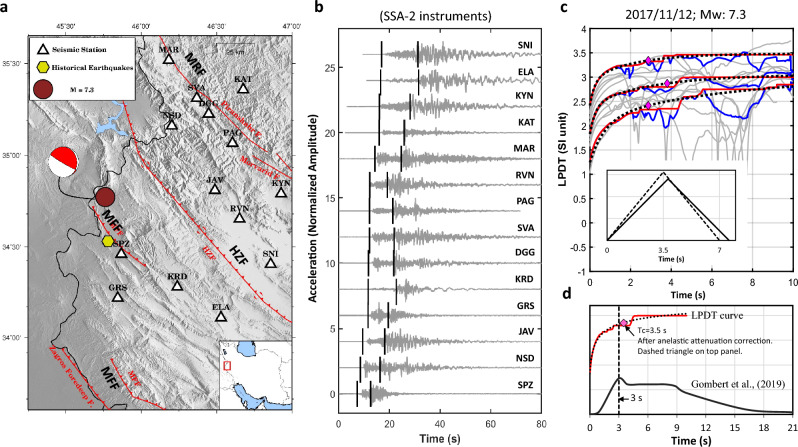
(**a**) Map shows the epicentre (red circle) of the largest event in the selected dataset, the 2017 Mw 7.3, Ezgeleh earthquake, and locations of the acceleration stations belonging to ISMN (triangles) deployed within 100 km epicentral distance. Yellow hexagons present the historical event^10^. (**b**) Vertical acceleration records, original data. The lines show the manual P- and S-wave arrival times. (**c**) The LPDT curve of the event (middle curve) and ± 1 standard deviation LPDT curves (top and bottom curves) (for more detail, see Zollo et al.^27^). The fitted curve to the LPDT curve (red curve) is shown with the black dotted line. The corner time of the plateau level is shown with a magenta diamond. Solid and dashed black triangles represent the source time function of this event before and after anelastic attenuation correction, respectively. The bottom plot compares the LPDT curve with trapezoidal source time function of this event obtained by Gombert et al.^24^.

The dataset contains seismic records within a hypocentral distance < 100 km; we require at least 4 available records for each event. Figure 1 shows the map of the selected earthquakes and the accelerometric stations belonging to the ISMN within the explored distance range, in addition to the distribution of the focal depth, magnitude, and moment magnitude versus hypocentral distance for the whole database. An individual map of the Ezgeleh earthquake is shown in Fig. 2a. Figure 2b displays the vertical acceleration waveform including the manual P- and S-wave arrival times (black-solid lines) for the stations within the 100 km epicentral distance. Noting that, for the entire dataset, the P- and S-wave arrival times are identified manually on the vertical and horizontal components of about 584 ISMN stations, respectively. To estimate the source parameters of the events using the LPDT method, a 0.075 Hz high-pass Butterworth filter is applied to the displacement records obtained by twice integrating the raw data (acceleration waveforms).

## Method

In this study, we have implemented a time-domain and average-based method called "LPDT" which analyzes the P-wave signals recorded by local accelerometer/velocimeter network sensors deployed in the near-source distance range of the earthquakes^28^. This method uses a P-wave displacement waveform integrated from the vertical component of the input data, i.e., acceleration or velocity. Once the displacement signals have been corrected by hypocentral distance (R) i.e., simply multiplication of amplitude by R, the traces are aligned with reference to the first P-wave onset. Then, the LPDT curve is built as the average value of the logarithm of distance-corrected displacement amplitude at any previously defined time windows (TW) from the first P arrival, in which the TW can be as small as time sampling of the waveforms. The whole P-wave signal from the vertical component is used excluding the S-waves based on an a priori known S-minus-P time window. Consequently, using only the P wave simplifies the rupture property computations without any sort of complexities associated with considering S waves or computing waveform spectra.

Figure 2c shows how the LPDT curve for the Ezgeleh earthquake (blue curve) is calculated by averaging distance-corrected displacement amplitudes (grey curves) obtained by double integrating the vertical acceleration waveforms recorded within 100 km of the epicentre (Fig. 2b). Following Zollo et al.^28^, a new curve is then built up by keeping the maximum amplitude (red curve, upper envelope in Fig. 2c) in the expanded time window from the P-onset. Finally, a three-parameter exponential function (dotted black curve) is best-fitted to the curve:

1 $$LPDT\left(t\right)={LPDT}_{0}+{P}_{L}\left(1-0.5\left({e}^{-\mathrm{t}/{T}_{1}}+{e}^{-\mathrm{t}/{T}_{2}}\right)\right)$$

where ${T}_{1}$, ${T}_{2}$ and ${P}_{L}$ are the parameters estimated by a nonlinear curve-fitting procedure with constraints (${T}_{2}$>${T}_{1}$), while ${LPDT}_{0}$ corresponds to curve value at the starting time of the curve, here set to t = 0, that correspond to the first P-wave arrival time. ${T}_{1}$ and ${T}_{2}$ have no specific physical meaning but are used to model different shapes of LPDT curves. Note that the plateau level equals to the ${P}_{L}^{*}={LPDT}_{0}+{P}_{L}$.

As clearly shown, the LPDT curve has a ramp-like shape with an initial rise, a concave curvature, and a maximum level (plateau). To obtain a reliable estimate of the corner time (${T}_{c}$) and its corresponding amplitude i.e., plateau level, the curvature of the fitted curve is evaluated to find a time at which the curvature tends to zero (magenta diamonds, Fig. 2c). As demonstrated in the previous studies, the amplitude at the corner-time of the LPDT curve corresponds to the peak amplitude (${P}_{d}={10}^{{P}_{L}^{*}}$) of the source time function (STF) and its corresponding time (Fig. 2 in Nazeri et al.^27^).

In principle, this average-based method assumes that rupture spreads radially on a circular fault at a constant velocity (${V}_{r}$) with an isosceles triangular-shaped (Fig. 2c) moment rate function, which seems sufficiently accurate to describe the source parameters of small-to-moderate events analyzed in previous articles^27,28,31^. Wang et al.^31^ suggested that the rectangle fault (Haskell model) can be more suitable than the circular rupture model in the estimation of rupture length of the large events (Ms > 6.7). Wang et al.^31^ analyzed 31 moderate-to-large earthquakes that occurred from 2007 to 2015 in China with Ms ranging between 4 and 8. In fact, Wang et al.^31^ stated that this model is only able to correctly reproduce the rupture process of the events with Ms ≤ 6.7, because of underestimated rupture length of the larger events in their dataset (Ms > 6.7) in comparison with the other studies. To this end, they assumed that the corner-time (${T}_{c}$) of the plateau level (${P}_{L}^{*}$) corresponds to middle point of the average trapezoid plateau. While, compared to Gombert et al.^25^ for the 2017 Ezgeleh earthquake, our ${T}_{c}$ estimation represents the rise time of the trapezoidal source time function (Fig. 2d).

This study only evaluates parameters of the circular source models after extracting the LPDT curve features, i.e., the corner-time (${T}_{c}$) and plateau level (${P}_{L}^{*}$), and then correcting for the anelastic attenuation effect by a post-processing procedure.

Independent to the rupture model, determination of the seismic moment (${M}_{0}$) relates to the area underneath the source time function (STF). In an elastic, homogeneous, and half-space Earth model, the far-field P-wave displacement radiated from a point-source rupture, ${M}_{0}$ is calculated via following formula^28,29,32^:

2 $${M}_{0}={{A}^{{^{\prime}}}}^{-1} {10}^{{P}_{L}^{*}} {T}_{c}$$

where ${{A}^{{^{\prime}}}}^{-1}$= $\frac{4\mathrm{\pi \rho }{{\mathrm{V}}_{\mathrm{p}}}^{3}}{{\mathrm{F}}_{\mathrm{S}}{\mathrm{R}}_{\mathrm{\vartheta \varphi }}}$ is a constant factor depending on the medium, $\uprho$ is density, ${\mathrm{V}}_{\mathrm{p}}$ is P-wave velocity, ${\mathrm{F}}_{\mathrm{s}}$ is the free surface factor, and ${\mathrm{R}}_{\mathrm{\vartheta \varphi }}$ is the average P-wave radiation pattern coefficient.

From the following equations, we can calculate the stress drop and source radius for a circular rupture^28,33^, in which ${V}_{R}$ is rupture velocity:

3 $$a={T}_{c}/\left(\frac{1}{{V}_{R}}-\frac{2}{{\pi V}_{P}}\right)$$

4 $$\Delta \sigma =\frac{7 {M}_{o}}{16 {a}^{3}}$$

Finally, average slip ($\overline{D }$) is obtained through the below formula in which $\mu$ is the shear modulus calculated from density ($\rho$) and shear wave velocity of the medium (${V}_{s}$) i.e., $\rho {V}_{s}^{2}$ and $A$ is area calculated as $\pi {a}^{2}$.

5 $${M}_{o}=\mu A \overline{D }$$

## Results

**Figure 3:**
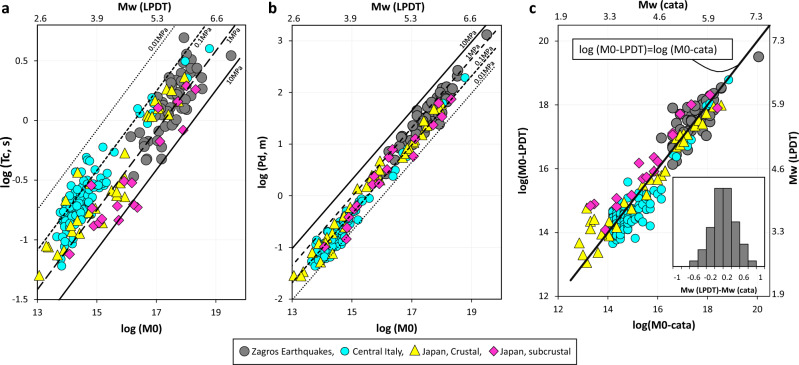
For comparison, the Zollo. et al.^27^ observations for Central Italy and Japan are plotted in all panels. (**a**, **b**) Peak amplitude (${P}_{d}$) of the STF and its corresponding time (${T}_{c}$) (dark grey circles) as a function of the seismic moment in the logarithm scale after applying the Q-filter assuming Q equals to 100. These plots include the theoretical scaling assuming four constant stress drop values of 0.01 MPa (black dotted line), 0.1 MPa (black dashed line), 1 MPa (black long-dashed line), and 10 MPa (black solid line). (**c**) Logarithm of the seismic moment obtained from the LPDT method as a function of values reported by ISMN. The black dashed line shows the one-to-one relation between the values. Histogram shows the magnitude estimation residual, difference between magnitude estimated using LPDT approach and ISMN catalogue values.

Figure 3a and b show the linear increase of the two parameters of the LPDT curves, i.e., ${T}_{c}$ and converted plateau level (${P}_{L}^{*}$ to peak amplitude (${P}_{d}={10}^{{P}_{L}^{*}}$), with the seismic moment in logarithmic scale. The larger the seismic moment is associated with the higher ${T}_{c}$ and ${P}_{d}$ values. In principle, ${T}_{c}$ and ${P}_{d}$ represent the half-duration and peak amplitude of the triangular moment-rate function after correcting for the anelastic attenuation. These Figures include the theoretical scaling of each parameter as a function of the seismic moment by fixing the stress drop to 0.01–10 MPa. Figure 3c compares the logarithm of the seismic moment obtained from the LPDT method, $\mathrm{log}\left({M}_{0}-LPDT\right)$, and those reported by BHRC, $\mathrm{log}\left({M}_{0}-cata\right)$, showing a good consistency between the values. It is worth noting that in BHRC, the moment magnitude is estimated in the frequency domain using the spectral method based on the Brune source model^34^ along with the Global Centroid Moment Tensor (CMT) catalog^35,36^. In this regard, the high-frequency spectral decay factor (γ) is fixed to 2, while the quality factor is calculated through the inversion of the displacement spectrum.

**Figure 4:**
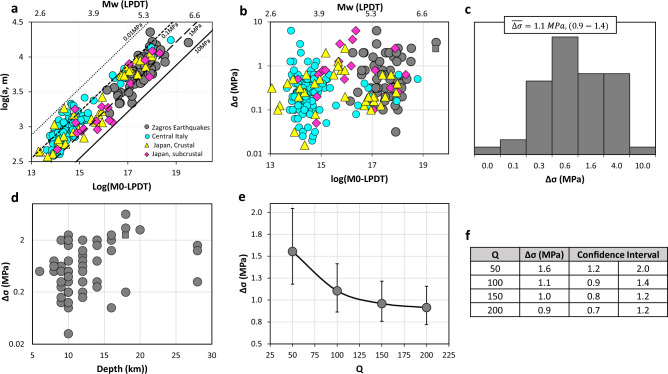
Circular rupture model. (**a**) Logarithm of the source radius, and (**b**) Stress-drop of the individual events versus the estimated seismic moment using the LPDT method. The theoretical scaling assuming four constant stress drop values of 0.01 MPa (black dotted line), 0.1 MPa (black dashed line), 1 MPa (black long-dashed line), and 10 MPa (black solid line) are presented as well. (**c**) Histogram of the stress-drop values. (**d**, **e**) Plot show the dependency of the stress-drop values to the depth and the average P-wave constant quality factor of the area, respectively. (**f**) Average stress-drop and a 95% confidence interval for each individual Q value. Panels a and b include the measurements by Zollo et al.^27^ for Japan and Italy. Panels b and d include solution of the stress drop value for the Ezgeleh earthquake assuming Haskell model, shown with dark grey rectangle.

Assuming the circular crack model, the linear increasing relation is observed for the source radius of the rupture versus the seismic moment in the logarithmic scale (Fig. 4a). Figure 4b presents the stress-drop parameter as a function of the logarithm of the seismic moment obtained from the LPDT method. While the variability of stress release with different ${Q}_{P}$ values is investigated in Fig. 4e.

**Figure 5:**
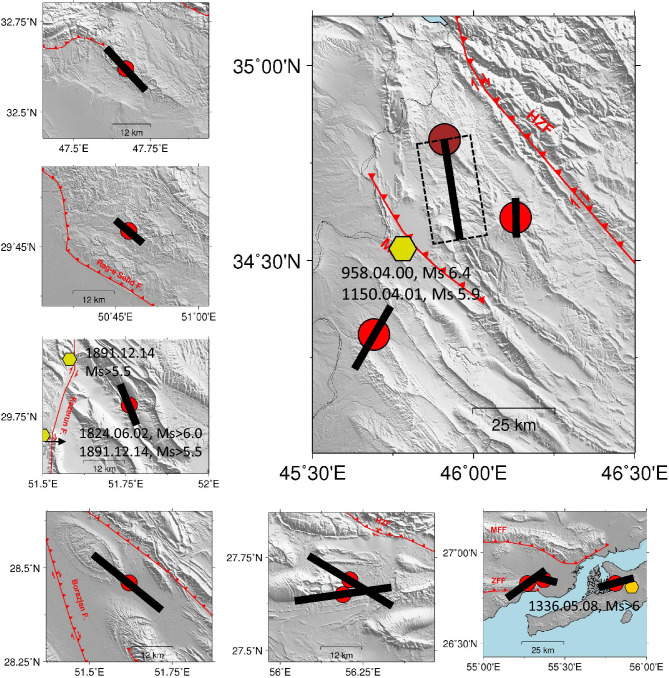
Segments including large events shown in Fig. 1 with rectangles. Rupture lengths are shown as oriented projections along the strike of the causative fault (see Table 1). For the Ezgeleh earthquake, dashed rectangle shows the surface projection of the fault obtained from the Haskell model. This figure also represents the available historical events^10^ with yellow hexagons at each segment.

Table 1 reports the source parameters of the events estimated in this study, while for the largest events with Mw ≥ 6, Fig. 5 represents the estimated fault-diameter segments oriented according to the strike direction of the potential causative fault as inferred from the focal mechanism. The historical seismicity^10^ suggests that some of the corresponding fault segments have the potential to produce a large earthquake of the same size as the analyzed dataset (Mw > 5.5).

**Table 1:** Source parameter obtained in this study.

Num	Date.time (YYYYMMDD-hhmmss)	Catalogue	LPDT-method				
		Mw	Mw		Δσ (MPa)	a (km)	Slip (cm)
1	20060330.193620.80	5.2	5.4 ± 0.02		0.3 ± 0.16	6.5 ± 1.15	3.75 ± 1.25
2	20060331.011702.30	5.9	5.7 ± 0.27		2.0 ± 0.10	4.4 ± 0.05	19.96 ± 6.65
3	20060331.115405.20	5.0	5.1 ± 0.26		0.8 ± 0.35	3.1 ± 1.96	5.60 ± 1.87
4	20080827.215238.00	5.8	5.8 ± 0.13		0.5 ± 0.30	7.9 ± 0.25	9.13 ± 3.04
5	20080910.110040.00	6.0	6.1 ± 0.12		0.5 ± 0.12	12.0 ± 0.33	13.74 ± 4.58
6	20100223.102554.90	5.4	5.3 ± 0.20		1.0 ± 0.01	3.7 ± 0.69	8.58 ± 2.86
7	20100514.184956.00	5.1	5.0 ± 0.47		0.6 ± 0.05	3.0 ± 1.03	4.33 ± 1.44
8	20100927.112247.90	6.1	5.8 ± 0.31		0.2 ± 0.03	11.4 ± 0.87	5.23 ± 1.74
9	20101126.123344.00	5.5	5.4 ± 0.16		0.5 ± 0.02	5.1 ± 1.19	5.88 ± 1.9
10	20110105.055549.30	5.4	5.0 ± 0.35		0.8 ± 0.10	2.8 ± 0.07	5.06 ± 1.69
11	20110305.112445.00	5.2	5.7 ± 0.03		0.8 ± 0.05	6.6 ± 0.32	11.96 ± 3.99
12	20120503.100937.50	5.5	5.7 ± 0.09		0.2 ± 0.08	10.0 ± 1.75	4.57 ± 1.52
13	20130112.032502.80	5.0	5.3 ± 0.07		0.1 ± 0.10	7.2 ± 0.57	2.08 ± 0.69
14	20130409.115249.10	6.0	5.9 ± 0.05		2.5 ± 0.08	5.7 ± 1.28	32.64 ± 10.88
15	20130409.120541.10	5.1	5.6 ± 0.18		0.1 ± 0.07	10.5 ± 0.05	2.42 ± 0.81
16	20130410.015827.00	5.7	5.5 ± 0.25		0.8 ± 0.10	5.2 ± 0.19	9.49 ± 3.16
17	20130506.022804.40	5.0	5.1 ± 0.09		2.0 ± 0.02	2.2 ± 0.33	10.01 ± 3.34
18	20131122.065124.40	5.6	5.6 ± 0.05		0.3 ± 0.09	7.6 ± 1.73	5.54 ± 1.85
19	20131122.183057.50	5.7	5.8 ± 0.04		1.6 ± 0.12	5.8 ± 1.38	21.07 ± 7.02
20	20131124.180542.80	5.5	5.5 ± 0.53		1.3 ± 0.08	4.1 ± 0.03	11.82 ± 3.94
21	20131128.135134.00	5.7	5.9 ± 0.30		0.5 ± 0.06	9.1 ± 0.32	10.46 ± 3.49
22	20140416.233140.00	5.0	5.0 ± 0.24		2.0 ± 0.30	2.1 ± 0.03	9.79 ± 3.26
23	20140818.023204.10	6.0	6.0 ± 0.10		1.6 ± 0.12	7.4 ± 0.07	26.83 ± 8.94
24	20140818.052551.20	5.8	6.1 ± 0.09		0.2 ± 0.08	14.9 ± 1.34	6.81 ± 2.27
25	20140818.112303.70	5.4	5.9 ± 0.35		0.1 ± 0.05	22.7 ± 0.25	1.64 ± 0.55
26	20140818.115133.00	5.8	5.9 ± 0.13		0.1 ± 0.08	16.5 ± 1.30	3.79 ± 1.26
27	20140818.180824.40	5.9	5.7 ± 0.12		0.5 ± 0.07	7.6 ± 0.30	8.70 ± 2.90
28	20140819.213216.40	5.4	5.6 ± 0.12		0.2 ± 0.08	9.0 ± 1.35	4.11 ± 1.37
29	20140820.101414.90	5.8	5.4 ± 0.07		6.3 ± 0.03	2.2 ± 1.92	31.55 ± 10.52
30	20140823.200518.80	5.5	5.6 ± 0.07		2.0 ± 0.10	4.2 ± 0.33	19.17 ± 6.39
31	20141015.133553.80	5.6	5.8 ± 0.25		0.3 ± 0.01	9.8 ± 2.18	7.11 ± 2.37
32	20141212.204542.10	5.0	5.9 ± 0.05		0.1 ± 0.09	14.1 ± 0.86	4.06 ± 1.35
33	20151125.211718.50	5.1	4.8 ± 0.17		2.0 ± 0.08	1.6 ± 0.25	7.13 ± 2.38
34	20171112.181816.20	7.3	6.9 ± 0.40	CM	3.4 ± 0.01	16.1 ± 2.06	125.86 ± 41.95
				HM	2.5 ± 0.02	*L*: 30.0 ± 1.25 *W*: 16.0 ± 2.12	206.86 ± 38.5
35	20171211.140957.00	5.4	5.4 ± 0.34		3.2 ± 0.10	2.7 ± 1.28	19.93 ± 6.64
36	20180106.152208.40	5.1	4.9 ± 0.44		0.3 ± 0.20	3.6 ± 0.54	2.09 ± 0.70
37	20180401.083525.70	5.2	5.4 ± 0.26		0.3 ± 0.08	6.2 ± 1.19	4.46 ± 1.49
38	20180825.221325.00	6.0	5.6 ± 0.03		0.6 ± 0.06	5.9 ± 0.49	8.52 ± 2.84
39	20181125.163731.80	6.3	6.3 ± 0.21		1.3 ± 0.01	10.5 ± 1.61	30.32 ± 10.11
40	20200609.171812.00	5.6	5.9 ± 0.18		1.3 ± 0.01	6.9 ± 1.40	19.83 ± 6.61
41	20200614.180558.00	5.0	4.7 ± 0.20		0.2 ± 0.01	3.4 ± 1.15	1.22 ± 0.41
42	20210217.183534.00	5.6	5.5 ± 0.18		0.6 ± 0.20	5.5 ± 0.38	7.92 ± 2.64
43	20210418.064150.00	6.0	5.7 ± 0.05		2.0 ± 0.10	4.7 ± 0.89	21.37 ± 7.12
44	20210718.143419.00	5.3	5.4 ± 0.33		0.3 ± 0.22	5.8 ± 0.54	4.20 ± 1.40
45	20211114.120704.00	6.1	5.9 ± 0.37		2.0 ± 0.05	5.8 ± 0.57	26.58 ± 8.86
46	20211114.120838.00	6.3	6.3 ± 0.41		2.5 ± 0.10	8.5 ± 0.97	48.69 ± 16.23
47	20220316.231544.00	5.9	6.0 ± 0.23		0.4 ± 0.08	11.5 ± 2.05	10.46 ± 3.49
48	20220615.060604.00	5.5	5.6 ± 0.70		0.2 ± 0.01	9.5 ± 1.40	3.45 ± 1.15
49	20220701.213206.00	6.1	6.0 ± 0.33		0.3 ± 0.01	12.6 ± 1.61	9.12 ± 3.04
50	20220701.232413.00	5.9	5.3 ± 0.03		1.6 ± 0.25	3.1 ± 0.93	11.29 ± 3.76
51	20220701.232514.00	6.1	6.2 ± 0.11		0.5 ± 0.20	12.6 ± 0.23	14.49 ± 4.83

Average slip is calculated assuming the shear modulus of $\rho {V}_{s}^{2}=3.2\times {10}^{10}Pa$, in which ${V}_{s}$ is average shear velocity that we used in this study. For the Ezgeleh earthquake, the source parameters assuming both circular (CM) and Haskell (HM) models are reported.

It is noted that all parameters estimated in this study for the Zagros area (dark gray circles) are compared with observations obtained by Zollo et al.^28^ for two other tectonic regions of Central Italy and Japan (Figs. 3 and 4). To estimate the earthquake source parameters, the required parameters are fixed as ${V}_{P}=6 \,  {\rm{km/s}}$, ${V}_{P}/{V}_{S}=1.75$, ${\alpha }^{{^{\prime}}}={V}_{R}/{V}_{S}=0.9$, ${\mathrm{F}}_{\mathrm{S}}{*\mathrm{R}}_{\mathrm{\vartheta \varphi }}=1$, and $\uprho =2.7 (\mathrm{kg}/{\mathrm{m}}^{3})$. The average values of ${V}_{P}$, and ${V}_{P}$-to-${V}_{S}$ ratio are obtained by referring to the work of Motaghi et al.^37^ and Talebi et al.^38^.

As for the anelastic attenuation correction, using a previously known P-wave, frequency independent quality factor for the region of concern, we refer to the study of Talebi et al.^39^, in which different attenuation factors are estimated using the extended coda normalization method by analyzing more than 6421 local events with a magnitude range of 3 to 5.5 that occurred from 2006 to 2019 within the studied area. Looking at the spatial variation of the quality factor, Talebi et al.^39^ concluded the higher attenuation in the northern parts of the Zagros region than in the southern part. Note that there is no overlap between this high-attenuation area and region including our dataset. According to their observation, the average ${Q}_{P}$ values of 93, 110, 165, 325, and 571 have been obtained at five central-frequencies of 1.5, 3, 6, 12, and 18 Hz respectively using data recorded within an epicentral distance of 100 km. Considering the magnitude range of the selected earthquakes (Mw ≥ 5.0) and analyzing the P-wave displacement spectra, the dominant corner frequency range does not exceed 6 to 7 Hz which corresponds to ${Q}_{P}$ values in the range 93–165 according to the study by Talebi et al.^39^. In our study, considering possible uncertainties on the estimation of ${Q}_{P}$, we decided to explore the source parameter sensitivity to seismic attenuation with ${Q}_{P}$ values ranging from 50 to 200, although Figs. 3, 4, and 5 present the result for the case ${Q}_{P}=100$.

## Discussion

The source duration, radius of the circular rupture, and stress drop obtained in this study show a consistent self-similar, constant stress drop scaling with magnitude, that is comparable with the theoretically expected trends (Figs. 3a and 4a).

Assuming a rupture velocity of 0.9 Vs, the average stress-drop value and its relevant 95% confidence interval vary between <Δσ>  = 1.6 (1.2–2.0) MPa to <Δσ>  = 0.9 (0.7–1.2) MPa for ${Q}_{P}$ values ranging from 50 to 200 (Fig. 4e,f). The average stress drop is in good agreement with the measurements of Raeesi et al.^8^ for the whole Zagros area which vary between 0.5 and 3 MPa. By applying different methods, Raeesi et al.^8^ concluded that the stress drop is a method-dependent parameter varying by one or two orders of magnitude, due to uncertainties in the rupture dimension estimation.

It is also worth noting that the stress drop depends on a variety of factors, such as the repeat time of earthquakes on a given fault segment^40^, fault mechanism^41^, and whether the earthquake is intraplate or interplate, shallow or deep^41,42^. In addition, rupture velocity plays a significant role in estimating source parameters since it is closely connected to the calculation of rupture radius and stress release. It has been shown by Longobardi et al.^30^ that the lower the rupture velocity, the higher the average stress release.

The choice of the rupture model (circular, unilateral, bilateral) and the rupture velocity applying frequency- or time-domain, parametric or not-parametric techniques is the primary assumption for the source parameter estimations, in particular the rupture length and area, from which the stress drop is derived. In spectral methods, rupture velocity is implicitly represented by a constant value in the inverse relation between the spectrum corner frequency and the source radius^44^. Brune^33^ assumed an instantaneous rupturing circular surface (infinite rupture velocity), while more realistic rupture velocities were introduced by the following studies of the dynamic expanding circular crack^43,45,46^ considering sub-shear rupture speeds in the range 0.5–0.9 ${V}_{S}$, depending on dynamic constraints of the adopted circular fault expanding model. Given the difficulties to estimate the rupture velocity from earthquake spectra (or time domain signals), almost all subsequent studies on source parameter scaling use either Brune^33^ or Madariaga^43^ relationships to estimate the source radius from the corner frequency, the latter being obtained for ${V}_{r}$ = 90% ${V}_{S}$. The Madariaga^43^ model provided equations for both P and S wave corner frequency while the Brune^33^ relationship was available only for S-wave corner frequencies.

In order to compare our rupture radius (and stress drop) estimates with similar measurements in other tectonic areas applying different methods, we use here the same rupture velocity value used in the Madariaga^43^ model, that are comparable to similar studies that used P-wave signals for source parameter estimations (e.g.,^47^).

In the case of the Ezgeleh earthquake’s rupture model, there is no agreement between the previous studies regarding the number and sizes of rupture asperities, the maximum amount of slip, and the rupture velocity as well. For example, Nissen et al.^23^ observed that the mainshock ruptured slowly (∼2 km/s, e.g., 60% of ${V}_{s}$) for the first 10–15 s. Using this low rupture velocity, our estimations of the source radius and static stress drop change by about 9 km (area of π × 9^2^ ≈ 255 km^2^) and 20 MPa respectively, that is highly inconsistent with the general observed scaling obtained for other events in the same area.

As for the Ezgeleh earthquake, assuming the circular rupture model, our measurements of source parameters fall within the scatter range and follow the scaling trend of the estimated source parameters with calculated seismic moment from the LPDT method, despite the observation of Wang et al.^33^ for large magnitude events (M > 6.7) that needed the assumption of a rectangular Haskell-type rupture model to retrieve the self-similar scaling. For the Ezgeleh earthquake, the LPDT method estimates a Mw of ~ 6.9 after applying the anelastic attenuation correction (Q = 100). The corresponding time to the peak amplitude of the isosceles triangular-shaped STF is about 3.5 s (Fig. 2) which predicts the source radius and static stress drop of about 16.1 km and 3.4 MPa, respectively. There is a good agreement between our estimation of the stress drop value and the area of the circular rupture (∼π × 16.1^2^ ≈ 810 km^2^) with those obtained by Nissen et al.^23^ i.e., (∼2.7–3.7 MPa) and (∼40 × 20 ≈ 800 km^2^), who exploited local, regional, and teleseismic data to characterize the Ezgeleh earthquake rupture. Based on their analysis, they suggest that this rupture involved an oblique (dextral-thrust) slip across a broad (∼40 × 20 ≈ 800 km^2^), gently dipping (∼15°) rupture plane.

Our applied time-domain method fails to capture the total duration of the rupture for the Ezgeleh earthquake, even though the source parameter estimation falls within the scatter range of the overall analysed data and follows the expected theoretical scaling. The noncomparability of the P-wave time window with rupture duration due to the use of near-source distance stations, as well as the rupture model, may lead to an underestimation of rupture duration/size. In case of an incomparable P-wave time window with rupture duration, there is a risk of not reaching the plateau and obtaining monotonically increasing the LPDT curve. As seen in Fig. 2, the LPDT curve of the Ezgeleh earthquake exhibits a plateau, so the second factor mentioned above, namely the underlying rupture model, is more likely to be the source for underestimating the rupture duration. In fact, this methodology has a significant limitation in that it assumes a circular rupture model and a triangular event source function, where the corner time (${T}_{c}$) is interpreted as the half-duration of the rupture. While, for the Ezgeleh earthquake, ${T}_{c}$ represents the rise time of the trapezoidal source time function. It means that the current assumption of this method does not adequately describe the complexity of a large event source, unless regional scale data is included, or the rupture model is modified. Thus, a rectangular, Haskell-type rupture could be an alternative source model to solve the problem. Note that in source parameter estimations of small to large-magnitude earthquakes, rupture models (circular or rectangular-Haskell type models) may result in different rupture lengths for an event with the same magnitude.

To explore possible errors in fault dimension and stress-drop estimates due to the circular fault model, we re-evaluate from the LPDT parameters, the earthquake fault parameters adopting the unilateral rectangular (Haskell) rupture model and the approach followed by Wang et al.^33^. Madariaga^43^ provided the relations between stress drop and average slip and rupture width (length, radius) for different rupture models (circular to linear) as $\Delta \sigma =\frac{1}{{c}_{\sigma }}\mu \frac{\langle \Delta u\rangle }{W}$; where μ is the rigidity, 〈∆u〉 is the average fault slip, W is the fault width and ${c}_{\sigma }$ is a coefficient depending on the rupture mode (${c}_{\sigma }$=16/7π for a circular fault; ${c}_{\sigma }$≅1 for a rectangular fault).

Assuming a long rectangular fault, the stress drop is calculated by, $\Delta \sigma =\frac{2}{\pi }\frac{{M}_{0}}{{W}^{2}L}$, where L and W are the length and width of the fault respectively. Wells and Coppersmith^48^ scaling law is used to calculate the corresponding width of the fault. While the same scaling law could be simply considered to obtain the length of the rupture, we prefer to calculate it by using the available solution on the LPDT curve, i.e., last point ($L={T}_{end} {V}_{r}$). This selection is made due to the continued increase of the amplitude of the curve showing the plateau part of the apparent trapezoidal STF. Hence, the rupture surface in this model is $L$ × $W$ ≈ 30 × 16 ≈ 480 km^2^ (dashed rectangle shown in Fig. 5) with an average static stress drop of 2.5 MPa, in line with the overall average stress drop.

In general, in the stress-drop estimates from different rupture models (e.g., near-circular or rectangular) using the above Madariaga’s formula and assuming the same rigidity and average slip, the difference in the results relies on the assumption of W and the constant coefficient of ${c}_{\sigma }$. Considering the empirical relations between subsurface rupture width and length inferred from Wells and Coppersmith^48^ one can estimate an L/W ranging between 1.5 and 2.9 (average 2.2) for earthquake magnitudes between M 6 and 7. So we expect that in this specific magnitude range, the stress drop for a rectangular fault is about a factor 1.4 larger than the value that we obtained assuming a circular fault model**.**

## Conclusion

A fast time-domain technique, based on the average of the P-wave displacement waveforms recorded within 100 km epicentral distance, is used to determine the source parameters of the large earthquakes (Mw ≥ 5.0) that occurred in the fold-and-thrust Zagros belt, one of the seismically active areas on the Iranian plateau. The scaling law of rupture dimension (radius/length) versus seismic moment, obtained in this study, coupled with a near-constant, self-similar stress-drop scaling with a median value of about 1 MPa would provide input data enabling the development and implementation of advanced earthquake simulation scenarios for moderate to large earthquakes in the Zagros region. This provides valuable results for seismic risk assessment, from the possibility of a combination of observed and simulated peak ground motion intensities.

To better understand how the methodology and the assumed circular rupture model depend on the seismic region/regime and the focal mechanism of the causative fault, the measurements of this work are compared with those of Zollo et al.^27^ for Central Italy and Japan seismic zones.

The main conclusions of our study are here summarized:

- Different applications of the proposed method for various tectonic areas such as Iran, Italy, The Geysers, China, and Japan in different magnitude and distance ranges confirm that this approach is valid for such a wide variety of data when using near-source, strong motion recordings. The algorithm is feasible for rapid and reliable source parameter estimation of moderate to large magnitude events from real-time streamed strong-motion signals collected by a dense accelerometric network deployed in the earthquake source region. The method complements well the source parameter determinations that for the M > 5.5 magnitude range are only available from regional and tele-seismic networks, several minutes after the event occurrence, but with the advantage to exploit high-frequency near-source signals emitted by generally complex earthquake ruptures.
- Furthermore, because of applying a consistent method across the whole dataset, this comparison allows us to discuss the regional dependency of the estimated source parameters, particularly the stress release. The Iranian earthquakes occurred within a compressive tectonic regime, while the earthquakes in Central Italy occurred within an extensional intraplate regime, and the Japanese catalogue of crustal and subcrustal events along a subduction zone. Although the self-similarity and constant stress drop are evident for whole data and individual regions, Central Italy earthquakes with a normal faulting mechanism exhibit a lower average stress drop of 0.36 (0.30–0.44) MPa. Also of interest is a comparison of our results with the Japanese catalogue that follows a very similar scaling but still different in average stress drop of 0.60 (0.42–0.87) MPa and 1.53 (1.01–2.31) for crustal and subcrustal events respectively. Furthermore, we do not observe a depth dependence of the stress release which is consistent with previous observation from comprehensive analysis of global P wave spectrums by Allmann and Shearer^41^.
- We found that our magnitude estimation is compatible with that of the ISMN catalog. As a result of computing the difference between the calculated magnitude in this study and those in the catalogue (obtained from spectrum of the S-wave), the residual shows a normal distribution (Fig. 3). The median is 0.02, while the first and third quartiles are 0.2 and 0.6, respectively. Noting that a combination of S and P waves may improve final estimates, however, it will also complicate signal processing and will strongly rely on data availability. Picking the S phase is more challenging than identifying the P phase, and there is a risk that a limited number of S-wave traces will be available in a given area.

Another benefit of using P-wave is that it is possible to use the same methodology for real-time applications, such as early warning systems, even though it is performed offline in this study. In conclusion, based only on measurements of P-wave peak amplitudes, the proposed method provides a straightforward, and simple approach for estimating earthquake source parameters.

## Data Availability

A The datasets analyzed during the current study are available in the Road, Housing & Urban Development Research Centre, Iranian Strong Motion Network (BHRC) repository, https://ismn.bhrc.ac.ir/en.
